# Screening of Phytoplankton Dynamics: Assessing Reservoir Ecosystem Health under Thermal Pollution from an Electrical Power Plant in the Pechora River Basin, European North

**DOI:** 10.3390/life14010071

**Published:** 2023-12-31

**Authors:** Elena Patova, Julia Shabalina, Michael Sivkov, Sophia Barinova

**Affiliations:** 1 Institute of Biology, Komi Scientific Centre, Ural Branch, Russian Academy of Sciences, Kommunisticheskaya St. 28, Syktyvkar 167982, Russia; patova@ib.komisc.ru (E.P.); julia-n-shabalina@rambler.ru (J.S.); sivkov@ib.komisc.ru (M.S.); 2 Department of Natural Sciences, Pitirim Sorokin Syktyvkar State University, Oktyabrsky Prosp., 55, Syktyvkar 167982, Russia; 3 Institute of Evolution, University of Haifa, Mount Carmel, 199 Abba Khoushi Ave., Haifa 3498838, Israel

**Keywords:** phytoplankton, diversity, bioindicators, water quality indices, thermal impact, Pechorskoe Reservoir, European North

## Abstract

For the first time, we investigated species composition, phytoplankton community structure, and hydrochemical parameters in the artificial cooling reservoir of a major thermal power plant (TPP) in northeastern Europe located in the Pechora River basin (Komi Republic). Our research, conducted during June and August, revealed a total of 81 species of algae and cyanobacteria, with cyanobacteria predominating. Among these cyanobacteria and microalgae (Bacillariophyta and Chlorophyta), algae that serve as reliable indicators of water quality were identified. The assessment of water quality based on abundance and species composition of indicator phytoplankton species revealed that the waters of the Pechorskoe Reservoir belong to the III class (β-mesosaprobic or moderately polluted). This indicates that water quality is satisfactory, and the reservoir retains the ability to self-purify. The power plant’s discharges heat the surface layers, increasing plankton communities’ diversity, abundance, and biomass. Such stable warming in the upper layers throughout the season, uncommon for natural water bodies in the north, results in a slight increase in the trophic status of the studied reservoir, supported by hydrochemical analysis. These results provide valuable information about ecosystem functioning under temperature increasing for predicting changes in the phototrophic biota of small northern reservoirs facing the impacts of climate change.

## 1. Introduction

Artificial water bodies, such as reservoirs, cooling-, fish-, and recreational ponds, have played a significant role in human economic activities since ancient times [1]. Monitoring the key ecosystem parameters, specifically, water quality and biological resources, is essential to assess waterbodies’ conditions, ensuring their preservation via thoughtful management decisions [2]. Together with physicochemical parameters, dynamic changes in the plankton community reflect the reservoir’s state and ongoing processes within it. Plankton plays a vital role in aquatic ecosystems, constituting a substantial portion of biomass entering the food chains [3]. In artificial reservoirs, an interplay between natural and human-induced factors causes dynamic alterations in the composition and production characteristics of plankton communities [4].

Comprehensive, long-term studies of the effects of human activities on plankton dynamics were previously conducted in large water reservoirs throughout various regions of Russia, such as the Basins of the Volga River [5,6], the Ob and Yenisei Rivers in Siberia [7,8], the Kama River [9,10,11], the Ural River [12], as well as in Primorsky Krai [13]. Moreover, extensive research on phytoplankton has been conducted in the Dnieper reservoirs and Danube nearshore area of Ukraine [14,15,16]. Worldwide, studies investigating the influence of temperature on phytoplankton have been carried out in reservoirs in the Republic of Belarus [17], China [4,18,19], and Israel [20].

Small water reservoirs and ponds, characterized by volumes of less than one million cubic meters and the absence of permanent spillways, often receive little attention in research and thus lack data regarding their biota and overall state. Despite their modest size, these water bodies together have a large positive impact on ecosystems, actively contributing to water regime regulation, purification of aquatic ecosystems, and the preservation of biological diversity globally [21]. Hence, it becomes crucial to understand how those systems function and further systematically monitor and evaluate their state.

In the Komi Republic, located in the northeast of European Russia, artificial reservoirs are predominantly small, with the largest ones mainly found in the southern part of the region in the Vychegda River basin. The planktonic communities of these reservoirs have been investigated, especially in recent years, providing valuable information on the composition and structure of aquatic invertebrates, including plankton [22].

Our research focused on the Pechorskoe Reservoir, which serves as a source of cooling water for a large power plant, setting it apart from other reservoirs in the study area. The continuous inflow of heated water from the power plant leads to persistent thermal pollution in this reservoir. The increased heat is known to elevate concentrations of biogenic substances, consequently raising the trophic level of the reservoir. In water bodies impacted by heated discharges, there is a noticeable increase in secondary pollution and a heightened presence of toxic substances [23].

Research on how thermal pollution impacts planktonic communities in small reservoirs in high northern latitudes is currently limited. Moreover, such data will not only help in developing effective strategies for monitoring, conservation, and sustainable resource usage but also deepen the understanding of the consequences on small water ecosystems in the north caused by rising temperatures in the northern hemisphere, as outlined in references [24,25,26]. The phytoplankton communities of the Pechorskoe Reservoir were studied for the first time to fill the lack of this information.

Our objective was to characterize the phytoplankton communities within a northern reservoir ecosystem under the influence of the discharge of warm water from a nearby power plant.

## 2. Materials and Methods

### 2.1. Description of the Study Site

**Figure 1:**
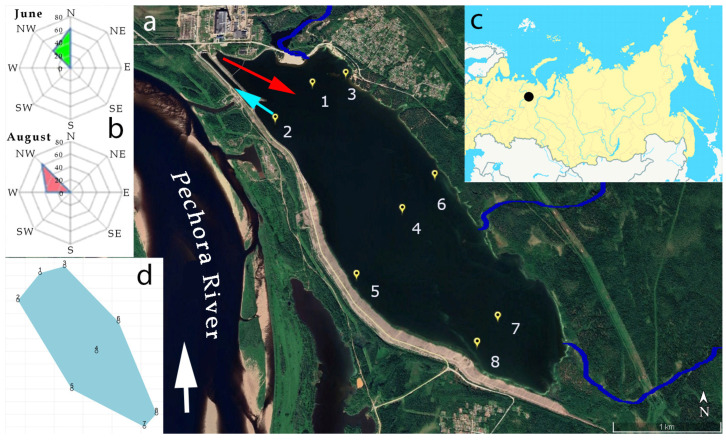
Location of the Pechorskoe Reservoir. Yellow pins (1–8) indicate sampling sites; a red arrow show location where warm waters are discharged, and a blue arrow marks the point where water enters the cooling system. (**a**)—a fragment of the satellite image (taken on 7 June 2022) with tributaries as blue lines; (**b**)—wind rose for June (green area) and August (rose area) 2022; (**c**)—the location of the reservoir on the map of northern Asia; (**d**)—schematic representation of sampling stations on the lake surface statistical map.

The study was conducted in the artificial reservoir Pechorskoe, situated in the middle reaches of the Pechora River basin, approximately 3 km from the town of Pechora (Figure 1). It was established in 1984 on the site of a previously existing swamp by merging two lakes and flooding the surrounding forests [27]. The reservoir is small and shallow, covering approximately 600 hectares, with around 33 million cubic meters in volume and a surface area of 5.74 square kilometers, as described in a reference [27]. The reservoir reaches a maximum depth of 7.6 m. Its coastline is linear, with the southern shore reinforced by concrete slabs. Three streams feed the reservoir. They flow into the reservoir from the northern side between stations 1 and 3 and 6 and 7 (Figure 1).

The power plant circulates 502,698 cubic meters of water annually from the lake, with an average temperature difference of 7.3 °C between the inlet and outlet throughout the year. Complete water exchange in the reservoir occurs within 15 days.

During summer, the water temperatures at the spillway and surrounding area range from 12 °C to 29 °C, while in the winter, they drop to 6 °C to 9 °C, with 30% of the area covered with ice. The Pechorskoe Reservoir has an average water temperature above 10 °C from mid-April to the end of October. This is two months longer than what was observed in other water bodies in the region throughout the years [28]. Rare temperature spikes, reaching up to 30–35 °C in the summer, have been observed in the reservoir; such occurrences are atypical for northern reservoirs [29]. Additionally, the reservoir is home to a relatively diverse fish population [28,29].

The movement of water masses in the reservoir is strongly influenced by the wind and air temperature. On the days when sampling took place in June, there were prevailing northerly to northwesterly winds with an average speed of 5 m/s and gusts reaching up to 10 m/s. On the day preceding the measurements, a southerly wind blew in the first half of the day; in the afternoon, it changed to a northwest wind of 3 m/s. In August, the prevailing wind direction was northwesterly to westerly, with an average wind speed of 3 m/s. On the day preceding the measurements, a northwest wind was blowing about 5 m/s. Data on wind direction during the study period were taken from the resource http://pogodaiklimat.ru (accessed on 15 January 2023). The average daily air temperature on 21 June was 17.5 °C, on 16 August—10.5 °C (https://www.meteorf.gov.ru/about/structure/cgms/3118/) (accessed on 15 January 2023).

It is possible that the distribution of warm water emissions across the water area of Pechorskoe Reservoir is influenced by the barrier structures of the power plant and features of the bottom topography.

### 2.2. Methods of Plankton Sampling and Hydrological Investigation

**Figure 2:**
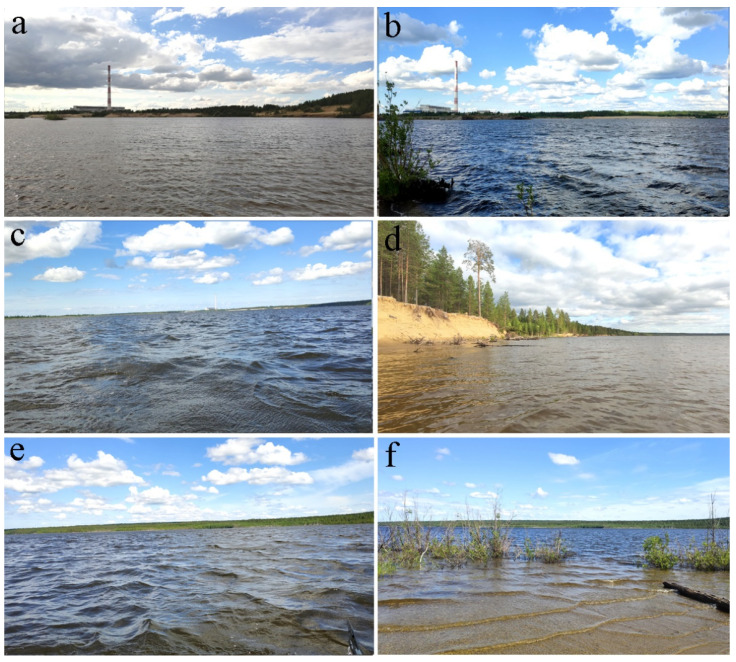
Photographs showing the sampling stations in the Pechorskoe Reservoir. (**a**)—st. 1. upper part of the reservoir, center; (**b**)—st. 2. upper part near the southern coast (place where warm water is discharged); (**c**)—st. 4. central part of the reservoir; (**d**)—st. 6. the central part of the reservoir on the north coast; (**e**)—st. 7. lower part, center; (**f**)—st. 8. lower part near the southern coast. Pictures by E. Patova.

In 2022, sampling for hydrobiological and hydrochemical analyses was conducted twice during the year at the Pechorskoe Reservoir. The period of ice-free water in the Pechora Reservoir lasts from early June to early September; ice is observed in the remaining months. The first set of samples was collected on 21 June, signifying the start of summer, and the second set was obtained on 16 August, indicating the end of the summer season. The sampling was comprehensive, covering eight various locations distributed evenly within the reservoir and located at the south, center, and north parts (Figure 1 and Figure 2, Table 1). The sampling was performed from the boat. A total of 16 hydrobiological samples were examined.

#### 2.2.1. Hydrochemical Samples and Water Temperature

**Figure 3:**
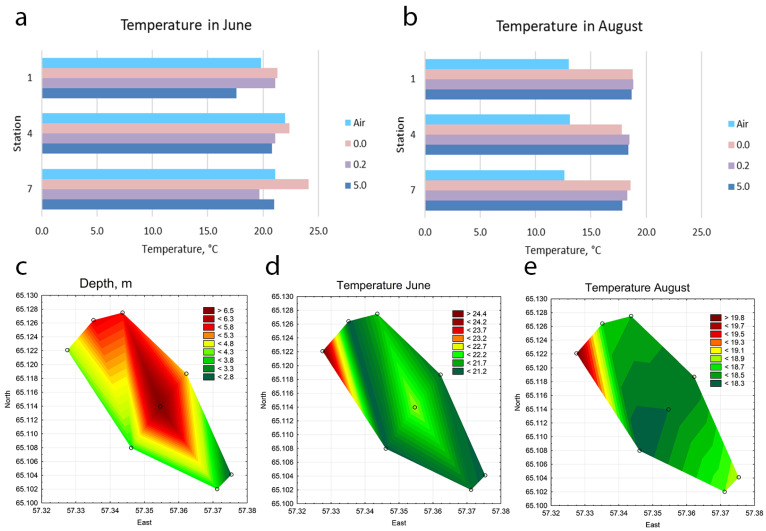
Temperature of water of the Pechorskoe Reservoir (at a depth of 0, 0.2, and 5 m) and air at the studied stations across the north–south axis of the reservoir on the sampling day; (**a**)—in June, (**b**)—in August. Statistical maps of depth (**c**) and water surface temperature in June (**d**) and August (**e**).

The bathymetric plan of the reservoir is given in Figure 3c. The water samples were collected following standard methods [30]. Surface water was directly sampled into 4.5 L glass bottles by submerging its mouth, while samples from 30 to 50 cm depth were collected using a 1.5 L Ruttner bathometer. Immediately after collection, the samples were placed in a cooling container to preserve their integrity. A comprehensive chemical analysis based on 26 water parameters was conducted in a certified analytical laboratory at IB KSC Komi SC UB RAS. It follows the requirements of ISO/IEC 17025:2017 (GOST ISO/IEC 17025-2019, https://www.iso.org/ru/standard/66912.html (accessed on 25 February 2019)) standards and has a quality certificate issued by the Register of Accredited CABs, number POCC RU.0001.511257. A more detailed description of the methods and analysis methods can be found in our previous publication [31].

Surface water temperature was measured with a multi-parameter analyzer HI98129 Combo (HANNA Instruments, Nusfalau, Romania). The bottom-water temperature and dissolved oxygen content were determined using a HACH LANGE HQ30D oxygen meter with an LDO oxygen sensor (HACH LANGE, Loveland, CO, USA). Furthermore, water temperature data from an environmental laboratory of the thermal power plant were used.

To compare the temperature regime of the Pechorskoe Reservoir with natural water bodies, the temperature of the streams feeding it was measured. Their temperature on 21 June 2022 was 12.3–12.7 °C, and on 16 August 2022 was 9.1–9.8 °C. The average daily temperature of the Pechora River based on measurements at the meteorological station (Ust-Kozhva meteorological station of Center for Hydrometeorology and Environmental Monitoring of the Komi Republic, https://www.meteorf.gov.ru/about/structure/cgms/3118/) (accessed on 15 January 2023) located a few kilometers from the Pechorskoe Reservoir was also used. The average daily water temperature in the Pechora River was 17.8 °C on 21 June 2022 and 14.5 °C on 16 August 2022.

#### 2.2.2. Phytoplankton Samples

To obtain phytoplankton samples, 50 L collected from surface layers were filtered through a plankton net (specifically, mill gas no. 77) [30]. Both live samples and samples fixed with a 4% formaldehyde solution were analyzed. For algae identification, two microscopes were used: the Nikon Eclipse80i, equipped with a differential interference contrast system and video camera (Nikon, Tokyo, Japan), and the Bimam-I (LOMO, St. Petersburg, Russia). Diatoms were identified in temporary, calcined, and permanent slides in Elyashev’s medium [30,31,32]. The species were identified following international keys ([30,33,34,35,36,37,38,39], and others), and taxonomic nomenclature follows current guidelines [40]. To correctly evaluate the abundance and biomass of phytoplankton, cells were directly counted in the Goryaev camera following standard protocol [30]. This method ensured the precise measurement of both the abundance and biomass of the phytoplankton species under study.

The mean and standard deviation for each data point were calculated based on five replicates using Microsoft Excel 2019+. This statistical approach enabled a comprehensive assessment of data variability and ensured the study’s consistency.

In terms of ecological categorization, the three most abundant/biomass species (up to five in cases of identical values) were designated as dominants, while the subsequent three (or four) species were classified as subdominants. This approach provides a systematic understanding of the phytoplankton community’s structure and composition.

### 2.3. Index Saprobity Determination

To obtain the saprobity index, a semi-quantitative method was implemented. Phytoplankton abundance is evaluated based on a 9-score-point system where each point represents the relative (percentage) abundance of individual taxa in the sample [41]: 1 point: less than 1%; 2 points: 2–3%; 3 points: 4–10%; 5 points: 11–20%; 7 points: 21–40%; and 9 points: 41–100%.

The water’s saprobity index is calculated based on the Pantle–Buck formula, modified by Sládeček [42]: S = Σhs/Σh, where S is the saprobic index of the phytoplankton community, s stands for the species-specific saprobic valence [42], and Σh is the sum of points on the 9-point scale.

The species ecology is described based on literature reviews [41]. The biological indicator parameter was assigned based on the species-specific preferences of algae and cyanobacteria for certain ecological conditions and water quality observed in [41,43]. Water quality classes were determined based on a comprehensive ecological classification of surface waters [41].

### 2.4. Statistical Analysis

To determine variation in the number of species in taxonomic phyla, standard deviation (stdev) was calculated in Excel for each distribution and reflected the value of the number of species that were expected around the mean. A low stdev indicates that the values tend to be close to the mean of the data set, while a high stdev indicates that the values are spread out over a wider range.

Statistical maps were generated using the Statistica 13.0 program, incorporating both parameter values and GIS coordinates of the sampling stations [43]. This method involved applying statistical mapping techniques to visualize the distribution of the variables. These maps are created for a wide range of chemical and biological indicators as well as calculated indices specific to reservoirs [43]. For this work, the maps of the distribution of the total species diversity, abundance, and biomass of phytoplankton, as well as index saprobity in the reservoir, were compiled. They supplement the obtained data with calculated values of the probable distribution of indicators of phytoplankton communities throughout the water area of the reservoir.

The maps were delineated based on the contour of the reservoir surface. This approach allowed for the inclusion of landscape features and the precise positioning of both specific points and diffuse influencing factors from the catchment basin into the analysis. To further refine the understanding of differences in the composition and abundance of phytoplankton communities, a correlation analysis was conducted using the JASP program [44]. This analysis aimed to uncover relationships and patterns within the data, providing valuable insights into the ecological dynamics of the reservoir.

## 3. Results

### 3.1. Characteristics of the Hydrochemical Parameters, Water Temperature, and Movement of Water Masses in the Investigated Water Bodies

Table 2 provides data on the hydrochemical parameters observed at sampling stations, showing that the conditions at the three stations during various sampling periods were quite similar. In general, the surface waters at all these stations exhibit relatively low mineralization, which is indicated by similar conductivity. The ion balance is primarily characterized by the dominance of hydrogen-carbonate ions and calcium ions, with comparatively low levels of chloride and sulfate ions, consistent with the hydrogen-carbonate water classification. Additionally, the concentrations of nitrate nitrogen, phosphates, ammonium, and other indicators were low (Table 2).

The temperature difference between water intake and water discharge is 6–9 °C in June and 5–6 °C in August, as the power plant laboratory report shows. Information on water temperature at both surface, 0.2 m depth, and bottom-water temperature, as well as air temperature recorded at the time of sampling, is presented in Table 1. Figure 3a,b show the ratio of water temperature at stations 1, 4, and 7 in the deepest part of the reservoir (Figure 3c) relative to air temperature at the time of sampling. In June, the water temperature at all stations was close to the air temperature. In August, the water temperature at the stations was higher than the air temperature. We also additionally used the power plant laboratory report with water temperatures at warm water discharge sites to construct an approximate diagram describing the distribution of water temperature in the reservoir on sampling days (Figure 3c–e). The diagrams show the zones of increased temperature at stations 1–2 in the northern part of the reservoir in the place where warm water descends. But the distribution of temperature gradients in the water area of the reservoir in different months is not uniform. This is due to the influence of wind. On the days when sampling took place in June, there were prevailing northerly to northwesterly winds with an average speed of 5 m/s and gusts reaching up to 10 m/s. In August, the prevailing wind direction was northwesterly to westerly, with an average wind speed of 3 m/s (Figure 1b). Wind patterns play a crucial role in affecting the movement and mixing of water masses in the reservoir (Figure 1b). Therefore, we do not see a sufficiently clear distribution of warm emissions across the whole reservoir (Figure 3c–e).

The water temperature in the Pechorskoe reservoir is higher than in the natural water bodies of the study area. So, in the streams feeding the reservoir water temperature during the period of our observations was lower by 8–12 °C in June and 8–10 °C in August. In the Pechora River flowing next to the Pechorskoe reservoir (Figure 1), the mean daily water temperature (according to meteorological station data) was also lower by 3–6 °C on 21 June 2022 and 3–5 °C on 16 August 2022. Temperature stratification is not observed in the reservoir during the summer period, as shown by bottom temperature measurements (Table 1), but the influence of hot water from thermal power plant emissions is clearly visible on the maps in June and August (Figure 3d,e).

### 3.2. Phytoplankton of Reservoir and the Indicators

In the summer of 2022, the phytoplankton community of the Pechorskoe Reservoir comprised 81 taxa of cyanobacteria (Cyanobacteria) and microalgae from 6 phyla. From identified 81 taxa, 11 taxa belonged to Cyanobacteria, 9 taxa to Euglenozoa, 32 to Bacillariophyta, 3 to Miozoa (Dinophyceae), 2 to Ochrophyta, 18 to Chlorophyta, and 6 to Charophyta (as detailed in Table 3 and Table A1). The structure of the phytoplankton community observed during two sampling periods is described in detail below for each station.

In June, 55 taxa of cyanobacteria and algae were present in phytoplankton, while in August, it dropped to 51 taxa (as detailed in Table 3 and Table A1). During both sampling periods, the highest species richness was observed among diatoms, followed by green algae, with cyanobacteria ranking third (Table 3). The diversity of other identified taxonomic groups was notably lower. In August, the number of diatom species decreased, while an increase in species in some other taxonomic groups was observed (Table 3). This indicates variations in phytoplankton composition via time and seasonal changes in dominant groups.

**Figure 4:**
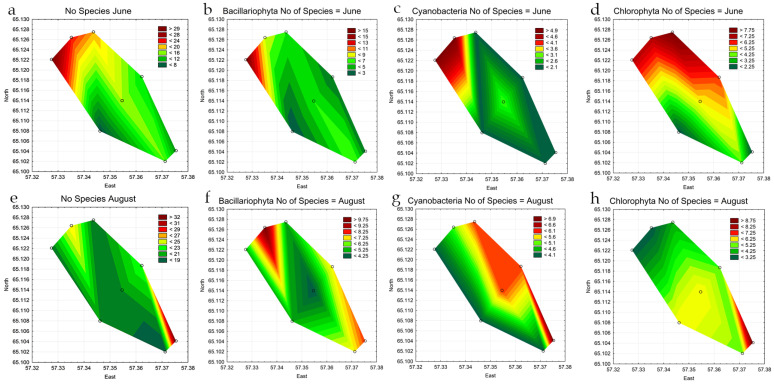
Statistical maps of the distribution of the total species diversity of phytoplankton (**a**,**e**) leading phyla: diatoms (**b**,**f**), cyanobacteria (**c**,**g**), and green algae (**d**,**h**) by stations of the Pechorskoe Reservoir in June–August 2022.

The trend in the distribution of species number across stations is shown on the statistical map in Figure 4a–h. In June, a larger number of species was observed in the northern part of the reservoir (st. 1–3), where the discharge of warm water occurs (Table 3). This area showed a higher diversity of cyanobacteria, diatoms, and green algae compared to the middle and southern parts of the reservoir. Such observation could be attributed to the fact that the reservoir’s waters warm up slowly after winter, and even a slight increase in water temperature triggers the rapid development of algae in warmed waters. By the end of August species number distribution across the surface layers of the reservoir becomes more uniform, compared to the beginning of the growing season. The number of diatom species continues to be the highest in the upper stations of the reservoir, and the diversity of cyanobacteria and green algae in the zone of warm water discharge remains approximately the same as in June but increases at other stations, with the highest increase in the coastal zones (Table 3, Figure 4e–h).

The distribution of dominant species across stations remained similar in June and August. Cyanobacteria, diatoms, and green algae groups showed the highest abundance (Table 3); these groups defined the ecological dynamics of phytoplankton in the reservoir.

**Figure 5:**
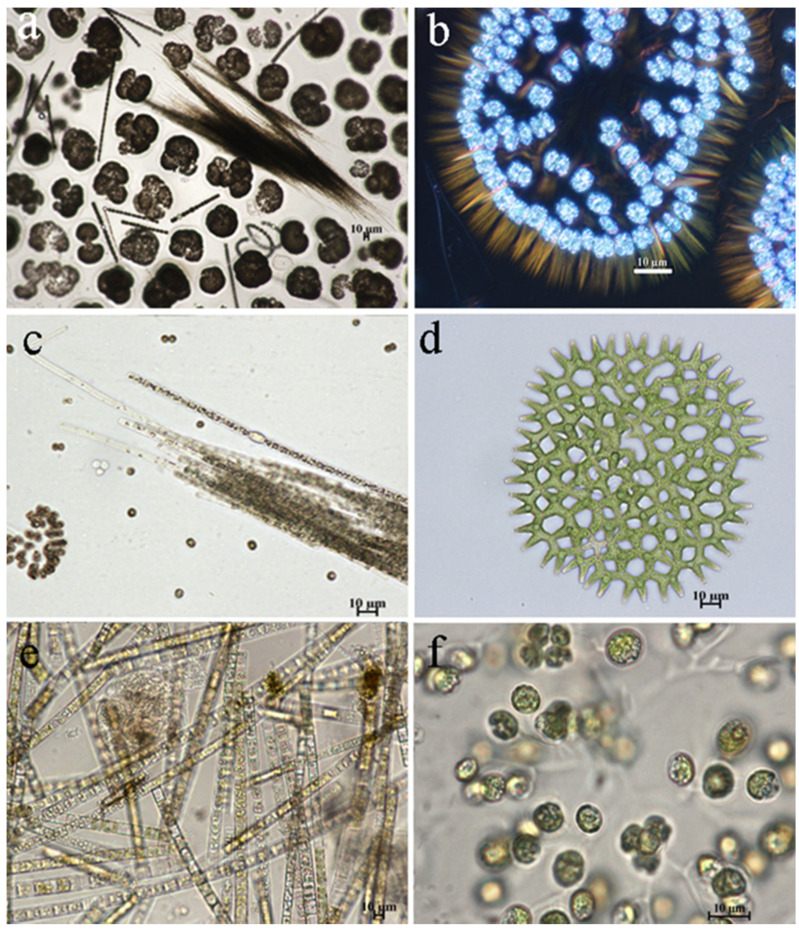
Dominant cyanobacteria and algae species in phytoplankton of the Pechorskoe Reservoir (June, August 2022): (**a**)—cyanobacterial water bloom; (**a**,**b**)*—Woronichinia naegeliana*; (**a**,**c**)—*Aphanizomenon flos-aquae*; (**d**)—*Pediastrum duplex*; (**e**)*—Aulacoseira italica*; (**f**)—*Hindakia tetrachotoma.* Pictures by E. Patova.

The species *Woronichinia naegeliana* dominated in all stations during both June and August, in numbers that could be characterized as “water blooming” (Figure 5a,b). The dominant species of phytoplankton community included diatoms such as *Aulacoseira italica* and *Fragilaria crotonensis*, as well as green algae *Hindakia tetrachotoma* and *Pediastrum duplex* (as shown in Figure 5d–f). In August, cyanobacteria species increased in abundance, such as *Aphanizomenon flos-aquae* (Figure 5e) and *Planktothrix agardhii*.

**Figure 6:**
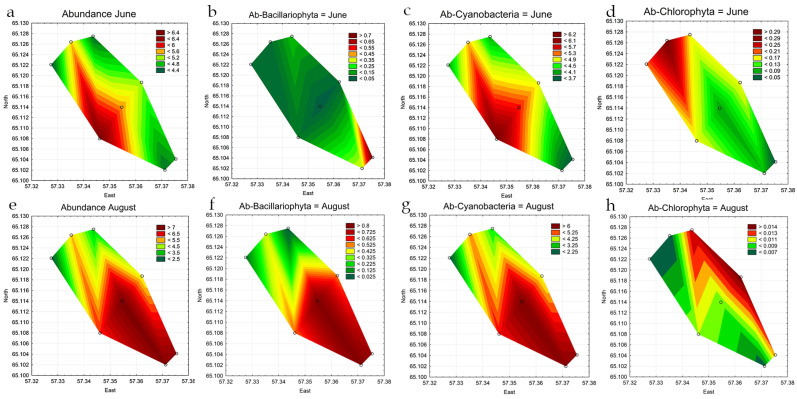
Statistical maps of the distribution of abundance (million cells L^−1^) of all phytoplankton groups (**a**,**e**) and leading phyla: Bacillariophyta (**b**,**f**), Cyanobacteria (**c**,**g**), and Chlorophyta (**d**,**h**) in the Pechorskoe Reservoir (June–August 2022).

The phytoplankton abundance varied widely across stations during observation periods, as detailed in Table 3. In June, the abundance ranged from 4.2 to 6.5 million cells L^−1^, while in August, the numbers ranged between 2.3 to 7.7 million cells L^−1^. The maximal abundance was observed in summer at station 4, which is located in the central part of the reservoir. Cyanobacteria were the dominant taxa at all stations in both June and August; other groups were present in substantially lower numbers (Table 3 and Figure 6).

The distribution of phytoplankton across the reservoir is heterogeneous (Figure 6a,e). The highest algae density in June was recorded at station 5 in the southwestern coastal part. As indicated in Figure 6c, cyanobacteria dominated the phytoplankton communities. Statistical analysis revealed that the concentration of cyanobacteria in the central part of the reservoir was significantly higher than in the upper (*p* = 0.037) and lower (*p* = 0.042) parts of the reservoir. Representatives of Bacillariophyta (Figure 6b) in June were distributed relatively evenly throughout the reservoir. In June, green algae were primarily concentrated at stations 1–3 in the upper part of the reservoir, particularly at the station where warm water discharge occurs (Figure 6d).

In August, a notable concentration of algae was observed in the central and lower parts of the reservoir, particularly at stations 4–8 (Figure 6e), which was confirmed by the analysis of variance (*p* = 0.016). Figure 6f,g illustrate that representatives of Bacillariophyta and cyanobacteria were concentrated in the central (*p* = 0.003) and lower (*p* = 0.003) parts of the reservoir, forming the core of phytoplankton abundance. In contrast, the concentration of green algae cells (Figure 6h) exhibited an opposite pattern, with maximum values for this group observed at stations 3 and 6 in the coastal zone of the reservoir.

**Figure 7:**
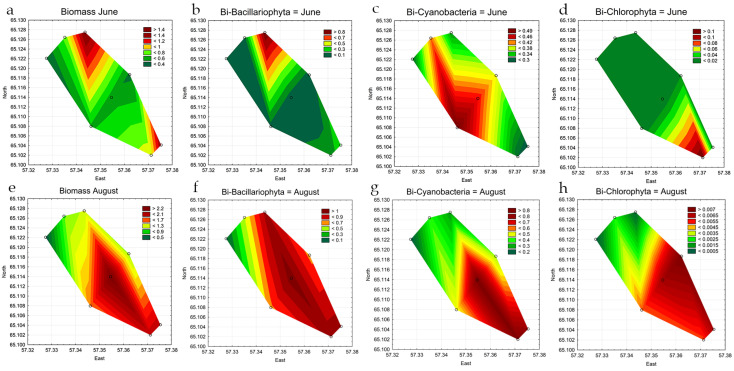
Statistical maps of the distribution of biomass (million cells L^−1^) of all phytoplankton groups (**a**,**e**) and leading phylum’s: Bacillariophyta (**b**,**f**), Cyanobacteria (**c**,**g**), and Chlorophyta (**d**,**h**) in the Pechorskoe Reservoir (June–August 2022).

The biomass of phytoplankton in June was lower than in August, with values ranging from 0.39 to 1.31 mg L^−1^ and 0.40 to 2.36 mg L^−1^ in August (Table 3). In June, the graphs of the projections of the biomass and abundance of plankton algae differ (Figure 6a and Figure 7a). The highest biomass was observed in the upper part of the reservoir at station 3, while the algal abundance was highest in the central part at station 5. Such discrepancy can be attributed to the cell size of the main biomass taxa. The high proportion of biomass in the upper part of the reservoir in June is formed by large-celled forms of diatoms (*Aulacoseira italica*, *A. granulata*, *Fragilaria crotonensis*). The highest biomass is associated with the development of large-celled dinoflagellata *Ceratium hirundinella* in the lower part of the reservoir at station 7. Also, small-celled cyanobacteria mainly contribute to abundance and biomass at most stations (Table 2, Figure 6c and Figure 7c).

Total phytoplankton biomass and diatom biomass have a similar distribution (Figure 7a,b). In August, the biomass graph is in good agreement with the graph of phytoplankton abundance (Table 2, Figure 6e and Figure 7e). Biomass, together with abundance, is noticeably higher in the central (st. 4 and st. 5) and lower (st. 7) parts of the reservoir than in the upper parts (st. 1–3) (*p* = 0.0021). The biomass indicators of individual groups of algae are consistent with the graphs of the distribution of the total biomass.

**Figure 8:**
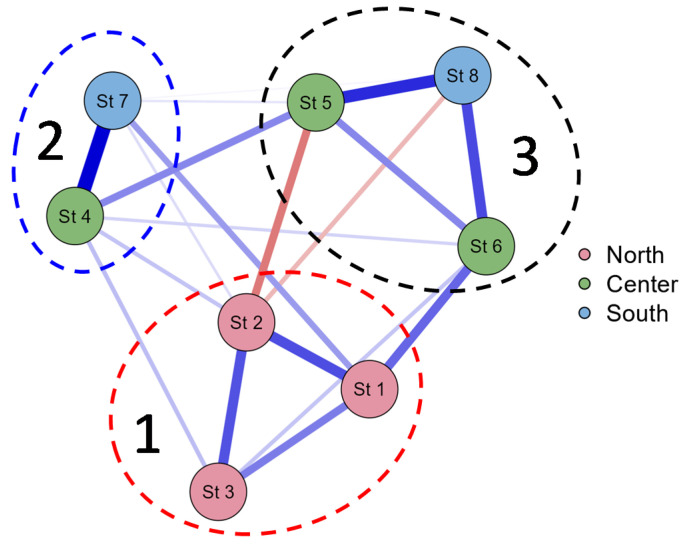
JASP correlation network plot for the phytoplankton communities of the Pechorskoe Reservoir by sampling stations. The strongest links are shown by the thickest lines. Positive correlations are shown in blue lines, and negative ones in red. Dashed lines outline clusters 1–3.

Figure 8 shows a JASP network diagram of correlations, and it can be seen that the phytoplankton communities of the Pechorskoe Reservoir could be grouped into three distinct clusters. The first cluster represents communities at stations 1–3, experiencing the greatest impact from warm water discharge. In the second cluster, stations 4 and 7 are grouped, representing phytoplankton communities in the central part of the reservoir, which is the deepest part of the reservoir, and the third cluster encompasses the plankton communities of the coastal zone of the reservoir.

### 3.3. Indicators of Habitat Conditions for Phytoplankton

**Figure 9:**
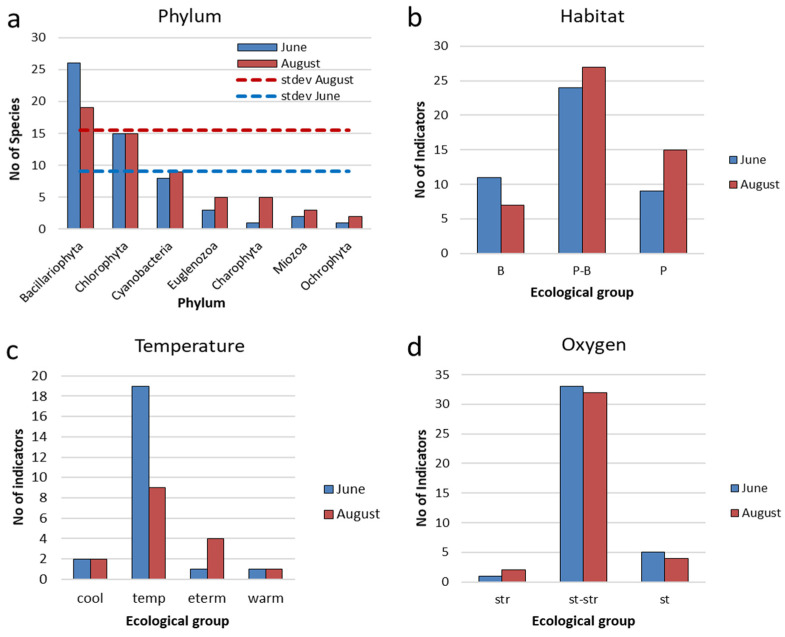
Distribution of taxonomic content ecological indicators (**a**), indicators of habitat (**b**), water temperature (**c**), and oxygen (**d**) for phytoplankton communities in the Pechorskoe Reservoir in June and August 2022. Habitat: P—planktonic; P-B—plankto-benthic; B—benthic. Temperature: cool—cool water; temp—temperate temperature; eterm—eurythermic; warm—thermophilic. Oxygenation and water moving: st—standing water; st-str—low-streaming water; str—fast-streaming water.

**Figure 10:**
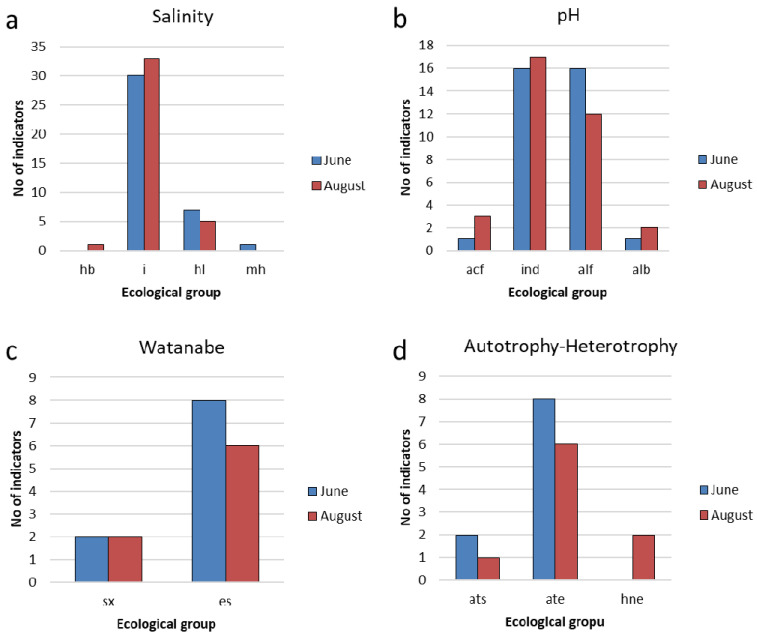
Distribution of indicators of salinity (**a**), pH (**b**), saprobity (**c**), and nutrition type (**d**) for phytoplankton communities in the Pechorskoe Reservoir in June and August 2022. Halobity degree (Salinity): hb—halophobes; i—oligohalobes–indifferent; hl—halophiles; mh—masohalobes. Acidity (pH): alf—alkaliphiles; ind—indifferent; acf—acidophiles; alb—alkalibiontes. Organic pollution indicators according to Watanabe: sx—saproxenes; es—eurysaprobes. Nitrogen uptake metabolism (autotrophy-heterotrophy): ate—nitrogen–autotrophic taxa; ats—tolerating elevated concentrations of organically bound nitrogen; hne—facultatively nitrogen–heterotrophic taxa, needing periodically elevated concentrations of organically bound nitrogen.

**Figure 11:**
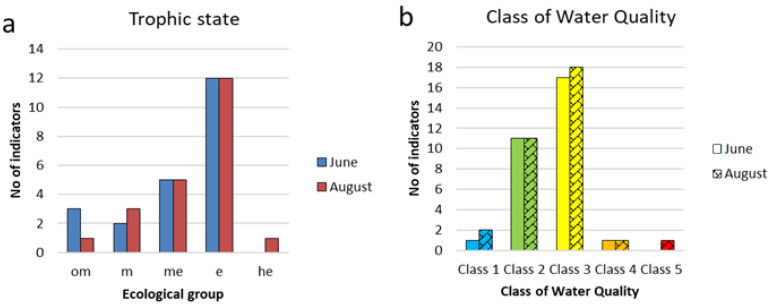
Distribution of indicators of trophic state (**a**) and class of water quality (**b**) for phytoplankton communities in the Pechorskoe Reservoir. Trophic state: om—oligo–mesotraphentic; m—mesotraphentic; me—meso–eutraphentic; e—eutraphentic; he—hypereutraphentic. The water quality class is determined as the sum of indicators whose species-specific index saprobity S from Table A1 is within the range of each class. Classes of water quality colored in EU color code (**b**).

The composition of indicator species in the Pechorskoe Reservoir appears highly similar between the two months (Table A1). Figure 9, Figure 10 and Figure 11 provide comparative histograms, which show the number of taxa and their corresponding environmental preference across various parameters in the Pechorskoe Reservoir. Among the organisms that are known as key indicators of various ecological conditions, the highest number of species was observed within diatoms and green algae (Figure 9a). During August, there was a higher number of species, which is shown by a higher stdev line and indicates that the values are spread out over a wider range.

In both June and August, phytoplankton communities were primarily formed by planktonic-benthic species (Figure 9b); toward the end of the summer season, there was a slight increase in planktonic species.

The algae and cyanobacteria species in Pechorskoe Reservoir primarily prefer a moderate regime of temperature (temp) or eurythermic (eterm). In June, the proportion of such species increases (Figure 9c). Only one thermophilic species (warm) was observed, *Ulnaria acus*, but with a low abundance. Four eurythermal species (eterm), primarily from the genus *Euglena*, were observed in August, although they were present with relatively low abundance.

Among the cryophiles (cool), the diatom *Aulacoseira italica* consistently appeared as a dominant species during both observation periods; *Aulacoseira italica* var. *tenuissima* was identified among the subdominant species.

These results show that the majority of the phytoplankton species in the Pechorskoe Reservoir prefer moderate temperature conditions. While there are a few species with a preference for either warmer or colder waters, they are not abundant in this ecosystem.

Indicators of the hydrodynamic conditions (oxygen regime and the mobility of water masses) are mainly represented by different species or species that prefer waters of moderate fluidity (st-str) [45]. The other groups are small (Figure 9d), but species found in stagnant waters are presented by dominant species—*Planktothrix agardhii*, *Woronichinia naegeliana*, and *Hindakia tetrachotoma*. The ratio of these groups remains the same throughout the studied period.

The salinity indicators [45] are dominated by diatoms. Indifferent species (i) are the most common, followed by halophiles (hl) (Figure 10a). Indifferent species were found in the phytoplankton composition at all studied stations. The composition of salinity indicators is typical for freshwater bodies with low salt content.

Based on pH, the species in the Pechorskoe Reservoir prefer slightly alkaline conditions to neutral (Figure 10b).

In June, indicators of oligosaprobic waters represented 94% in abundance, while the proportion of indicator species of β-saprobic conditions was about 3%. In August, indicators of oligosaprobic waters formed about 68% of the abundance and were dominant. The proportion of oligo-alpha-mesosaprobionts increased to 17%, indicating an increase in the trophic status of water bodies. Ecological saprobity types, according to Watanabe [45,46], are known only for a third of diatom species (Figure 10c). Of these, saprophytes predominated, among which were species of the dominant complex from the genus *Aulacoseira*. Saproxenes are rare; eurysaprobes were not observed. Figure 10d confirms that the identified species used an autotrophic mode of nutrition.

Among the trophic groups, eutraphentes (e) dominate during both periods and have half abundance as meso-eutraphentes (em) (Figure 11a). These two groups include dominant species. Mesotraphentes (m) are represented by 2–3 species. A slight increase in trophicity in August may be indicated by a decrease in the number of oligo-mesotraphentes (o-m) (three species in June and one in August) and the appearance of the highly eutrophic water species *Stephanodiscus minutulus* (he). Bioindication of the Water Quality Class is represented in Figure 11b and shows the prevalence of species of Class 3 in both the June and August sampling periods.

### 3.4. Statistical Mapping

**Figure 12:**
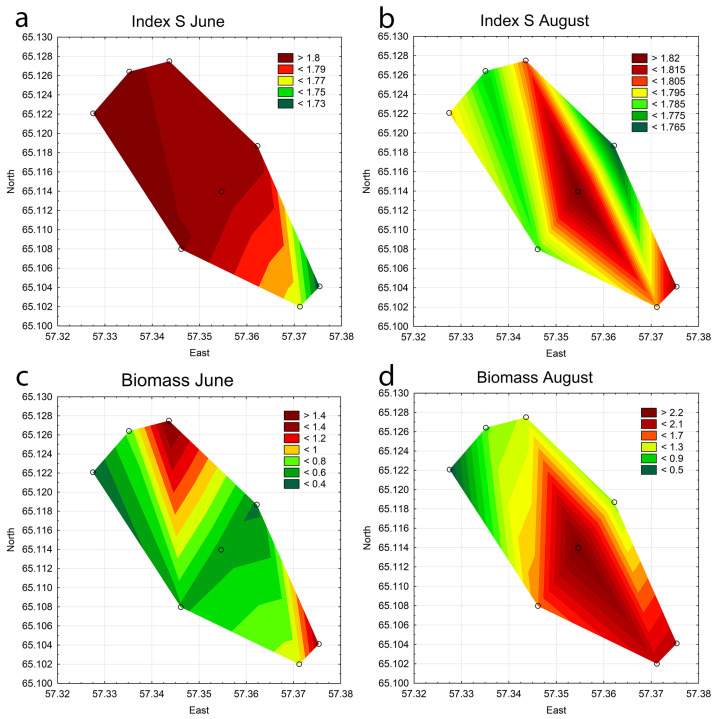
Combined statistical maps for the Pechorskoe Reservoir. Index saprobity S in June (**a**) and August (**b**); Biomass in June (**c**) and August (**d**). Colors are given for the value amplitude of each mapped variable from green (lower boxes in the legend key) to red (upper boxes in the legend key).

For visualization, statistical mapping was applied. For analysis, several maps were produced to represent a complex system of interactions between various indicators in all stations in the Pechorskoe Reservoir. For the current analysis, several maps were selected, showing the distribution of the most critical parameters (Figure 12).

The maps show that in June, the upper part of the Pechorskoe Reservoir had the highest phytoplankton biomass, influenced by the influx of warm water from the nearby power plant (Figure 12c). The effect was reduced in August (Figure 12d). During this time, probably water mixing due to wind played a more significant role in the algae concentration in the reservoir. Additionally, at stations 1–4, apart from the thermal power plant’s warming effect, the growth of planktonic organisms could be influenced by organic pollution from the stream that feeds the reservoir. This is indicated by an increase in the S index, especially in spring (Figure 12a,b). Nevertheless, the S index values for the entire study period at all stations fell within the range of 1.72–1.82, classifying the water quality belonging to class III, water of satisfactory quality, moderately saprobic, with remaining ability to self-purify [41].

## 4. Discussion

In the summer of 2022, the Pechorskoe Reservoir exhibited a diverse phytoplankton community comprising 81 taxa from 7 phyla (Table 3 and Table A1). Diatoms and green algae were the most species-rich groups, with cyanobacteria ranking third. The abundance and biomass of these phytoplankton organisms ranged from 2.3 to 7.7 million cells per liter and 0.39 to 2.36 milligrams per liter, respectively. Species composition and quantitative data are similar to those commonly observed in northern ecosystems of the study area [22,47] and northern water bodies of other regions [48].

Undoubtedly, the diversity of phytoplankton communities in natural water bodies, taking into account different ecological groups, is much higher. The Pechora River basin encompasses various water bodies, including the studied reservoir, which hosts 976 algal taxa from 10 divisions. In the past, in smaller man-made reservoirs in the same region, 300 species of algae and cyanobacteria were found, with abundance ranging from 0.23 to 5.48 million cells per liter and biomass from 0.45 to 0.98 milligrams per liter [22]. The biodiversity of phytoplankton in Pechorskoe Reservoir is comparable to other communities commonly found in the temperate zone and aquatic ecosystems with neutral pH [49,50].

The distribution of algae in the water surface of the Pechorskoe Reservoir is influenced by a combination of natural factors, such as wind patterns, the presence of shallow areas, bank erosion, the inflow of streams into the reservoir, as well as human activities related to the continuous discharge of warm waters. These factors collectively shape the composition and structure of phytoplankton communities. Therefore, showing the specific impact of elevated water temperature on phytoplankton communities is challenging. It is a well-established fact that water temperature plays a crucial role in governing the growth and reproduction rates of most planktonic algae. Most phytoplankton species achieve their maximum growth rate at around 20 °C [51]. An increase in water temperature above 25 °C leads to an increase in the maximum growth rate of cyanobacteria, and, as a result, they outnumber other phytoplankton groups [51].

This explains the fact that under conditions of constant inflow of heated water into the reservoir, already at the very beginning of the growing season, at all stations of the Pechorskoe Reservoir, a high diversity and biomass of cyanobacteria, as well as diatoms and green algae, was noted (Table 3). As measurements of water temperature in the bottom layers have shown, the Pechorskoe Reservoir is homothermic. This is a condition in which the temperature is the same in the whole volume of water, which affects the rapid development of phytoplankton, especially in spring. Usually, in June, for this latitude in lakes and reservoirs, phytoplankton development is very weak, as water in reservoirs is rather cold and warms up very slowly, and temperature stratification is common [52]. The influence of warm waters is also indicated by the presence of thermophilic species *Ulnaria acus* and four eurythermal species (Table A1). This species has been reported in a dominant complex in other reservoirs with thermal water pollution [53]. The most thermophilic species belong to the Euglenozoa phylum. They were found mainly in August in different parts of the reservoir but had low abundance.

The influence of thermal pollution on the species of phytoplankton was also shown by JASP correlation analysis (Figure 8), algae communities of st. 1–3 united into a separate cluster. These are the communities of sites that are most affected by warm water input. We also associate the dominance of the cyanobacteria *Woronichinia naegeliana* at all observation stations with the influence of warm waters. The mass development of this species in northern water bodies, resulting in “blooming”, is quite rare [54,55,56], but cyanobacteria blooming is known even in the permafrost zone water bodies [57]. Usually, at the beginning of the growing season, species from Bacillariophyta dominate the starting of the growing season of northern water bodies [47,48,49,50]. Diatoms are known to prefer colder water and are abundant in temperate lakes and reservoirs in autumn and spring when water temperatures are cooler [58]. It should also be noted that in all the observed periods, Bacillariophyta predominates in terms of the number of species in the Pechorskoe Reservoir; in August, their number decreases but remains quite high. While for many other reservoirs and cooling ponds, the predominance of Chlorophyta and an increase in the species richness of Bacillariophyta in the autumn period are usually noted [10,11,48,49,50]. In general, the discharge of heated water from the power plant does not significantly affect the composition and abundance due to the presence of various species adapted to different water temperatures.

Another factor that caused the difference in the distribution of abundance and biomass of algae in the Pechorskoe Reservoir is the strength and direction of the wind. During the sampling period in June and August 2022, a strong northern, northwestern wind was observed, which drove water masses to the central and south-western parts of the reservoir (st. 4–5, st. 7–8) (Figure 1). This factor primarily affected the total number of algae, which increased (Figure 4a,e) in the direction of the wind (Figure 1b). An increase in biomass at stations 4–6 was observed only for cyanobacteria, with total algae biomass reaching a maximum in the northeastern part of the reservoir at station 3 (Figure 7). The difference in biomass can be attributed to the substantial growth of planktonic-benthic diatoms and green algae in the northeastern part of the reservoir. These algae formed large, dense colonies; such a phenomenon was observed in other northern lakes as well [58].

The northern and northeastern shores of the Pechorskoe Reservoir experience erosion, and the coastal areas here are relatively shallow, with streams flowing into the reservoir in these areas. All of these factors likely contribute to water turbulence, increased water temperature, elevated nutrient levels, and consequently, result in rapid development of planktonic-benthic species. A common planktonic species with gas vacuoles, *Woronichinia naegeliana*, an undeniable dominant in the entire reservoir, can be transported by water currents during strong winds to the southwestern part of the Pechorskoe Reservoir at stations 4–8 (Figure 6g and Figure 7g).

Similar patterns have been observed in larger reservoirs. Lake Baikal, for instance, showed a connection between the origin of inflowing water masses and chlorophyll concentrations, which varied based on the direction of the wind [59]. In marine environments, mesoscale eddy dynamics have been noted to impact the productivity of marine systems, leading to changes in abundance, biomass, and chlorophyll concentration. However, the influence of eddy dynamics on the species composition of planktonic organisms remains less explored, making it difficult to comprehensively explain the observed changes in algal species composition under the influence of eddy dynamics [60].

The dominant phytoplankton species in the Pechorskoe Reservoir are mainly indifferent to salinity and pH, a common finding in the northern water bodies with neutral pH and low salinity (low salt content) [48,49,50]. The distribution of these indicator groups across different reservoir stations is consistent, indicating homogenous hydrochemical conditions in the reservoir. The reservoir’s trophic level, as indicated by algae, falls within the mesotrophic category. The Saprobity index values (Figure 12a,b) indicate that the ecosystem effectively manages both external and internal organic pollution. The effect of increasing water temperature on the development and species composition of phytoplankton and water blooming was studied in detail using bioindication and statistical mapping of parameters for various cooling reservoirs of thermal and nuclear power plants. The most striking example, similar to our findings for the Pechora reservoir, was a study of the influence of warm water emissions and eutrophication for two closely located reservoirs in the south of the European part [61]. However, the similarity of these processes was first identified for a reservoir in a cold climate zone in the north of European territory.

## 5. Conclusions

For the first time, algological studies were carried out in a small artificial northern reservoir, which experiences a constant influx of warm water from the power plant (TTP). The results of the study showed that phytoplankton communities during observation time did not experience significant structural and qualitative changes despite human activity. The effect of thermal pollution is more pronounced at earlier times of phytoplankton development. The assessment of water quality according to the species composition and abundance of phytoplankton species showed that the waters of the Pechorskoe Reservoir belong to the III class of water quality with satisfactory water quality purity, beta-mesosaprobic zone, with the capacity to maintain its cleanliness and self-purify. Based on the data obtained, it can be concluded that the reservoir ecosystem at present is in stable condition and has a self-regulating system despite the anthropogenic impact of the thermal power plant. The results obtained can be used to make forecasts of changes in the phototrophic biota of small northern water bodies under the conditions of climate change.

## Figures and Tables

**Table 1:** Descriptions of the sampling stations at the Pechorskoe Reservoir in June and August 2022, including coordinates and measurements for water pH and dissolved oxygen at the 0.2 m surface horizon.

Station	Location in Reservoir	Latitude	Longitude	Depth, m	Water Temperature *, °C June	Water Temperature *, °C August	pH June	pH August	Oxygen, mg O_2_ dm^−3^ June	Oxygen, mg O_2_ dm^−3^ August
1	An upper part, center	65.126408°	57.335149°	5.8	$\frac{{21.1\;}^{**}}{18.9}$	$\frac{18.6}{18.1}$	7.2	7.6	8.51	8.25
2	Upper part, near the southern coast (place of discharge of warm waters)	65.122078°	57.327509°	4.3	24.5	19.9	7.2	7.6	8.43	8.77
3	Upper part, on the north coast	65.127499°	57.343625°	5.7	24.5	18.5	7.2	7.9	8.60	8.85
4	A central part of the reservoir	65.113962°	57.354654°	6.9	$\frac{22.4}{21.4}$	$\frac{{17.8\;}^{**}}{17.5}$	7.2	7.7	8.50	8.86
5	The central part, near the southern coast	65.107971°	57.346128°	4.0	21.1	18.2	7.1	7.9	8.74	9.18
6	Central part, on the north coast	65.118693°	57.362238°	5.0	21.3	18.3	7.1	7.9	8.71	8.96
7	The lower part, center	65.104101°	57.375376°	3.9	$\frac{21.3}{21.0}$	$\frac{18.8}{18.6}$	7.1	8.1	8.61	9.58
8	The lower part, near the southern coast	65.102001°	57.371222°	2.8	21.1	18.9	7.1	8.1	8.82	9.60

Note: * the water temperature in the table represents specific measurements at the time of sampling. ** in the numerator, denotes the water temperature at the surface; in the denominator, at bottom-water temperature.

**Table 2:** Mean value of chemical parameters in Pechorskoe Reservoir in June and August 2022 with standard deviation.

	Station	1	4	7	1	4	7
Variable	Unit	June	June	June	August	August	August
pH	-	7.2 ± 0.2	7.2 ± 0.2	7.1 ± 0.2	7.6 ± 0.2	7.7 ± 0.2	8.1 ± 0.2
Electrical conductivity	µS cm^−1^	79 ± 4	78 ± 4	78 ± 4	87 ± 4	87 ± 4	89 ± 4
HCO_3_	mg dm^−3^	43 ± 5	43 ± 5	69 ± 8	47 ± 6	45 ± 5	48 ± 6
Total alkalinity	mmol dm^−3^	0.71 ± 0.09	0.70 ± 0.08	1.14 ± 0.14	0.77 ± 0.09	0.73 ± 0.09	0.79 ± 0.09
PV (Permanganate value)	mg dm^−3^	5.7 ± 0.6	5.9 ± 0.6	5.9 ± 0.6	6.3 ± 0.6	6.3 ± 0.6	6.3 ± 0.6
COD	mgO dm^−3^	21 ± 6	22 ± 7	18 ± 5	20 ± 6	21 ± 6	20 ± 6
CI^−^	mg dm^−3^	1.8 ± 0.3	1.9 ± 0.3	1.9 ± 0.3	1.61 ± 0.29	1.61 ± 0.29	1.80 ± 0.3
SO_4_^2−^	mg dm^−3^	4.5 ± 0.8	3.8 ± 0.8	3.8 ± 0.8	3.9 ± 0.8	4.1 ± 0.8	3.7 ± 0.8
PO_4_^3−^	mg dm^−3^	<0.05	<0.05	<0.05	<0.05	<0.05	<0.05
TSS	mg dm^−3^	0.0	1.45 ± 0.26	0.63 ± 0.14	1.17 ± 0.21	1.06 ± 0.19	2.00 ± 0.40
Fe	mg dm^−3^	<0.050	<0.050	<0.050	<0.050	<0.050	<0.050
Cu	µg dm^−3^	<1	6.1 ± 1.4	4.2 ± 1.0	5.3 ± 2.2	5.1 ± 2.1	4.4 ± 1.9
Phenol	µg dm^−3^	<0.25	0.35 ± 0.14	0.28 ± 0.11	<0.25	<0.25	<0.25
Zn	µg dm^−3^	<5	<5	<5	<5	<5	<5
Sr	µg dm^−3^	40 ± 10	41 ± 11	35 ± 9	41 ± 11	41 ± 11	42 ± 10
Mn	µg dm^−3^	1.7 ± 0.4	<1.0	1.3 ± 0.4	<1.0	<1.0	<1.0
Ni	µg dm^−3^	<1.0	<1.0	<1.0	2.6 ± 1.1	2.1 ± 0.9	2.0 ± 0.8
Ca	mg dm^−3^	10.0 ± 1.6	10.2 ± 1.6	9.05 ± 1.4	12.7 ± 2.0	12.6 ± 2.0	12.8 ± 2.1
Mg	mg dm^−3^	2.3 ± 0.3	2.3 ± 0.3	2.0 ± 0.3	2.6 ± 0.4	2.7 ± 0.3	2.7 ± 0.4
K	mg dm^−3^	0.86 ± 0.21	0.82 ± 0.20	0.73 ± 0.18	0.83 ± 0.20	0.83 ± 0.20	0.87 ± 0.21
Na	mg dm^−3^	3.2 ± 0.5	3.3 ± 0.5	3.0 ± 0.4	3.2 ± 0.5	3.2 ± 0.5	3.3 ± 0.5
N-NO_3_	mg dm^−3^	<0.010	<0.010	<0.010	0.018 ± 0.008	<0.010	0.015 ± 0.008
N-NO_2_	mg dm^−3^	<0.010	<0.010	<0.010	0.016 ± 0.006	<0.010	<0.010
N-NH_4_	mg dm^−3^	0.010 ± 0.002	0.011 ± 0.002	0.041 ± 0.009	0.061 ± 0.014	0.031 ± 0.007	0.070 ± 0.016
S_total_	mg dm^−3^	1.7 ± 0.4	1.8 ± 0.4	1.6 ± 0.4	1.8 ± 0.4	1.8 ± 0.4	1.8 ± 0.4
P_total_	mg dm^−3^	<0.020	<0.020	<0.020	0.027 ± 0.011	<0.020	<0.020

Note: <—marks values below the detection range.

**Table 3:** Indicators of phytoplankton development of the Pechorskoe Reservoir at the studied stations—number of species, abundance, biomass, and saprobity indices in June and August 2022.

Variable	St. 1	St. 2	St. 3	St. 4	St. 5	St. 6	St. 7	St. 8
No. of species (June)	23	30	20	16	7	13	15	10
No. of species (August)	26	19	19	20	20	23	18	33
Bacillariophyta, no. species June	8	16	6	6	2	3	7	3
Chlorophyta, no. species June	7	7	8	5	2	6	4	2
Cyanobacteria, no. species June	5	5	2	3	2	2	2	2
Euglenozoa, no. species June	1	0	3	0	0	0	0	1
Miozoa, no. species June	2	1	1	1	0	1	1	1
Ochrophyta, no. species June	1	1	0	1	0	1	1	1
Charophyta, no. species June	0	0	0	0	1	0	0	0
Bacillariophyta, no. species August	10	5	5	4	5	7	7	8
Charophyta, no. species August	3	2	3	1	2	1	1	4
Chlorophyta, no. species August	3	3	4	6	6	5	5	9
Cyanobacteria, no. species August	5	4	6	6	4	6	4	7
Euglenozoa, no. species August	2	3	0	1	1	1	0	2
Miozoa, no. species August	2	2	1	2	2	2	1	2
Ochrophyta, no. species August	1	0	0	0	0	1	0	1
Total Abundance, million cells L^−1^ June	5.5	4.33	4.47	5.60	6.49	5.13	4.48	4.68
Total Abundance, million cells L^−1^ August	5.30	2.29	3.54	7.74	6.01	4.90	7.61	7.24
Bacillariophyta, million cells L^−1^ June	0.08	0.05	0.17	0.03	0.08	0.05	0.34	0.74
Chlorophyta, million cells L^−1^ June	0.3	0.20	0.17	0.08	0.16	0.16	0.09	0.04
Cyanobacteria, million cells L^−1^ June	5.1	4.05	4.12	5.47	6.25	4.89	3.78	3.65
Bacillariophyta, million cells L^−1^ August	0.37	0.07	0.01	0.84	0.53	0.53	0.85	0.76
Chlorophyta, million cells L^−1^ August	0.01	0.01	0.01	0.01	0.01	0.02	0.01	0.01
Cyanobacteria, million cells L^−1^ August	4.88	2.20	3.47	6.78	5.40	4.31	6.64	6.33
Total Biomass, mg L^−1^ June	0.73	0.39	1.43	0.55	0.58	0.47	0.86	1.31
Total Biomass, mg L^−1^ August	1.08	0.40	1.23	2.36	1.60	1.15	1.96	1.99
Bacillariophyta, mg L^−1^ June	0.07	0.04	0.98	0.03	0.06	0.04	0.13	0.30
Chlorophyta, mg L^−1^ June	0.01	0.02	0.02	0.01	0.01	0.02	0.11	0.02
Cyanobacteria, mg L^−1^ June	0.42	0.30	0.32	0.44	0.50	0.39	0.30	0.29
Bacillariophyta, mg L^−1^ August	0.50	0.08	0.92	1.05	0.67	0.67	0.96	0.95
Chlorophyta, mg L^−1^ August	<0.00	<0.00	<0.00	0.01	0.00	0.01	0.01	0.01
Cyanobacteria, mg L^−1^ August	0.39	0.18	0.28	0.85	0.50	0.43	0.84	0.78
Index S June	1.81	1.81	1.80	1.81	1.81	1.81	1.76	1.72
Index S August	1.78	1.80	1.80	1.82	1.78	1.76	1.80	1.82

## Data Availability

The data presented in this study are available upon request from the corresponding author.

## Appendix A

**Table A1:** Ecological preferences of phytoplankton species of the Pechorskoe Reservoir in June–August 2022.

No.	Taxon	June	August	HAB	T	OXY	HAL	pH	D	SAP	Index S	AUT-HET	TRO
	Bacillariophyta												
1	*Acanthoceras zachariasii* (Brun) Simonsen	1	1	P	-	st-str	i	ind	-	o-b	1.4	-	-
2	*Achnanthidium minutissimum* (Kützing) Czarn.	1	1	P-B	eterm	st-str	i	ind	es	b	0.95	ate	e
3	*Asterionella formosa* Hassall	1	1	P	temp	st-str	i	alf	sx	b	1.35	ate	me
4	*Aulacoseira granulata* (Ehrenberg) Simonsen	1	1	P-B	temp	st-str	i	alf	es	b	2.0	ate	e
5	*Aulacoseira italica* (Ehrenberg) Simonsen	1	1	P-B	cool	st-str	i	ind	es	b	1.45	ate	me
6	*Aulacoseira italica* var. *tenuissima* (Grunow) Simonsen	1	1	P-B	cool	st-str	i	ind	es	b	1.3	ate	me
7	*Diatoma moniliformis* (Kützing) D.M.Williams	1	1	P-B	temp	st-str	i	alf	-	x-o	0.4	-	-
8	*Diatoma tenuis* C.Agardh	1	0	P-B	temp	st-str	hl	alf	-	b-a	2.4	-	om
9	*Encyonema cespitosum* Kützing	1	0	B	temp	st	i	alf	es	o-b	1.4	-	-
10	*Encyonema silesiacum* (Bleisch) D.G.Mann	1	1	B	temp	st-str	i	ind	-	-	-	-	-
11	*Entomoneis ornata* (Bailey) Reimer	0	1	B	-	st-str	i	alf	-	b	2.0	hne	-
12	*Epithemia sorex* Kützing	1	0	B	temp	st-str	i	alf	-	-	-	-	-
13	*Fragilaria crotonensis* Kitton	1	1	P-B	temp	st-str	i	alf	-	-	-	-	-
14	*Gomphonella olivacea* (Hornemann) Rabenhorst	1	0	B	temp	st-str	i	alf	-	b	2.3	ate	om
15	*Gomphonema acuminatum* Ehrenberg	1	0	B	temp	st-str	i	ind	-	x-b	0.8	-	-
16	*Gomphonema truncatum* Ehrenberg	1	0	B	temp	st-str	i	ind	-	b	2.0	-	-
17	*Gyrosigma acuminatum* (Kützing) Rabenhorst	1	1	B	temp	st-str	i	alf	-	-	-	-	-
18	*Iconella splendida* (Ehrenberg) Ruck & Nakov	0	1	P-B	-	st-str	i	alf	-	-	-	-	-
19	*Luticola mutica* (Kützing) D.G.Mann	1	1	B,S	temp	st-str	hl	ind	-	o-a	1.9	ats	e
20	*Navicula cryptotenella* Lange-Bertalot	1	0	P-B	temp	st-str	i	ind	-	-	-	-	-
21	*Navicula reinhardtii* (Grunow) Grunow	1	0	-	-	-	-	-	-	-	-	-	-
22	*Neidiomorpha binodis* (Ehrenberg) M.Cantonati, Lange-Bertalot & N.Angeli	0	1	B	-	str	i	alf	-	o	1.0	-	-
23	*Nitzschia frustulum* (Kützing) Grunow	1	0	P-B	temp	st-str	hl	alf	es	a-o	2.7	-	-
24	*Nitzschia gracilis* Hantzsch	1	0	P-B	temp	st-str	i	ind	-	-	-	-	-
25	*Nitzschia sigma* (Kützing) W.Smith	1	0	B	temp	st-str	mh	alf	-	-	-	-	-
26	*Rhopalodia constricta* (Brébisson) Krammer	1	0	B	-	st-str	hl	alf	-	-	-	ats	e
27	*Stephanodiscus hantzschii* Grunow	1	1	P	temp	st-str	i	alf	sx	-	-	-	-
28	*Stephanodiscus minutulus* (Kützing) Cleve & Möller	0	1	P	temp	st-str	i	alb	es	a-o	3.6	hne	he
29	*Surirella gracilis* Grun.	0	1	B	-	st-str	i	ind	-	-	-	-	-
30	*Tabellaria fenestrata* (Lyngb.) Kütz.	0	1	P-B	-	st-str	i	ind	-	o-a	1.9	-	-
31	*Ulnaria acus* (Kützing) Aboal	1	1	P-B	warm	st-str	i	alf	es	o-a	1.85	ate	me
32	*Ulnaria ulna* (Nitzsch) Compère	1	0	P-B	temp	st-str	i	alf	es	b-a	2.4	ate	e
	Charophyta												
33	*Closterium* sp.	1	0	-	-	-	-	-	-	-	-	-	-
34	*Cosmarium bioculatum* var. *depressum* (Schaarschmidt) Schmidle	0	1	P-B	-	st-str	hb	ind	-	x-o	0.5	-	m
35	*Mougeotia* sp. ster.	0	1	-	-	-	-	-	-	-	-	-	-
36	*Spirogyra* sp. ster.	0	1	P-B	-	-	-	alf	-	-	-	-	-
37	*Spondylosium planum* (Wolle) West & G.S.West	0	1	P-B	-	-	i	ind	-	-	-	-	-
38	*Staurodesmus triangularis* (Lagerheim) Teiling	0	1	P	-	-	i	acf	-	-	-	-	-
	Chlorophyta												
39	*Chlamydocapsa planctonica* (West & G.S.West) Fott	1	1	P-B	-	-	-	alf	-	o	1.2	-	-
40	*Coelastrum astroideum* De Notaris	0	1	P	-	st-str	-	-	-	b	2.2	-	e
41	*Coelastrum microporum* Nägeli	1	1	P-B	-	st-str	i	ind	-	b	2.3	-	e
42	*Desmodesmus communis* (E.Hegewald) E.Hegewald	1	1	-	-	-	-	-	-	-	-	-	-
43	*Eudorina elegans* Ehrenberg	0	1	P	-	st-str	i	-	-	b	2.3	-	-
44	*Eutetramorus globosus* Walton	1	1	-	-	-	-	-	-	-	-	-	-
45	*Hindakia tetrachotoma* (Printz) C.Bock, Pröschold & Krienitz	1	1	P	-	st	i	-	-	b	2.3	-	-
46	*Lemmermannia tetrapedia* (Kirchner) Lemmermann	0	1	P-B	-	st-str	i	ind	-	b	2.0	-	e
47	*Oedogonium* sp.	1	0	-	-	-	-	-	-	-	-	-	-
48	*Pandorina morum* (O.F.Müller) Bory	1	1	P	-	st	i	-	-	-	-	-	m
49	*Pediastrum duplex* Meyen	1	1	P	-	st-str	i	ind	-	-	-	-	e
50	*Pseudopediastrum boryanum* (Turpin) E.Hegewald	1	1	P-B	-	st-str	i	ind	-	b	2.1	-	e
51	*Pseudopediastrum boryanum* var. *longicorne* (Reinsch) Tsarenko	1	1	P-B	-	st-str	-	-	-	b	2.1	-	e
52	*Scenedesmus ellipticus* Corda	1	1	P-B, S	-	st-str	-	-	-	b-o	1.7	-	-
53	*Stauridium tetras* (Ehrenberg) E.Hegewald	1	1	P-B	-	st-str	i	ind	-	-	-	-	om
54	*Tetradesmus lagerheimii* M.J.Wynne & Guiry	1	0	P-B	-	st-str	i	ind	-	b	2.15	-	e
55	*Thelesphaera alpina* Pascher	1	1	B	-	str	-	-	-	-	-	-	-
56	*Ulothrix zonata* (F.Weber & Mohr) Kützing	1	0	P-B	-	st-str	i	ind	-	o-a	1.8	-	-
	Cyanobacteria												
57	*Aphanizomenon flos-aquae* Ralfs ex Bornet & Flahault	1	1	P-B	-	-	hl	alb	-	o-a	1.95	-	m
58	*Aphanothece* sp.	0	1	-	-	-	-	-	-	-	-	-	-
59	*Calotrix* sp.	1	1	-	-	-	-	-	-	-	-	-	-
60	*Chroococcus minimus* (Keissler) Lemmermann	0	1	P-B	-	-	hl	-	-	-	-	-	e
61	*Dichothrix ramenskii* Elenkin	1	0	-	-	-	-	-	-	-	-	-	-
62	*Dolichospermum smithii* (Komárek) Wacklin, L.Hoffmann & Komárek 2009	0	1	P	-	-	-	-	-	-	-	-	-
63	*Leptolyngbya* sp.	1	0	-	-	-	-	-	-	-	-	-	-
64	*Microcystis aeruginosa* (Kützing) Kützing	1	1	P-B	-	-	hl	acf	-	b	2.2	-	me
65	*Oscillatoria* sp.	1	1	-	-	-	-	-	-	-	-	-	-
66	*Planktothrix agardhii* (Gomont) Anagnostidis & Komárek	1	1	P-B	-	st	hl	-	-	-	-	-	-
67	*Woronichinia naegeliana* (Unger) Elenkin	1	1	P	-	st	-	-	-	o-a	1.8	-	e
	Euglenozoa												
68	*Astasiidae* cf.	1	0	-	-	-	-	-	-	-	-	-	-
69	*Euglena* sp.	0	1	-	-	-	-	-	-	-	-	-	-
70	*Euglena texta* (Dujardin) Hübner	0	1	P	eterm	st-str	-	ind	-	a-o	2.9	-	-
71	*Phacus* sp.	1	1	-	-	-	-	-	-	-	-	-	-
72	*Trachelomonas hispida* (Perty) F.Stein	0	1	P-B	eterm	st-str	i	acf	-	b	2.2	-	-
73	*Trachelomonas* spp.	1	0	-	-	-	-	-	-	-	-	-	-
74	*Trachelomonas volvocina* (Ehrenberg) Ehrenberg	0	1	P-B	eterm	st-str	i	ind	-	b	2.0	-	-
	Miozoa												
75	*Ceratium hirundinella* (O.F.Müller) Dujardin	1	1	P	-	st-str	i	-	-	o	1.3	-	e
76	*Peridiniaceae* sp.	0	1	-	-	-	-	-	-	-	-	-	-
77	*Peridiniopsis quadridens* (F.Stein) Bourrelly	1	1	P	-	-	-	-	-	o-b	1.4	-	-
	Ochrophyta												
78	*Dinobryon sertularia* Ehrenberg	1	1	P-B	-	-	i	-	-	o	1.3	-	-
79	*Tribonema vulgare* Pascher	0	1	P-B	-	-	i	-	-	o-b	1.4	-	-

Note: “0”—not recognized. Abbreviation of ecological groups. Habitat (HAB): P—planktonic; P-B—plankto-benthic; B—benthic; S—soil. Temperature (T): cool—cool water; temp—temperate temperature; eterm—eurythermic; warm— thermophilic. Oxygenation and water moving (OXY): st—standing water; st-str—low-streaming water, str—fast-moving water. Salinity ecological groups according to Hustedt (1938–1939) [62] (HAL): hb—oligohalobes-halophobes, i—oligohalobes-indifferent, hl—halophiles; mh—mesohalobes. Water pH preferences groups according to Hustedt (1957) [63] (pH): alf—alkaliphiles; ind—indifferent; acf—acidophiles; alb—alkalibiontes. Organic pollution indicators according to Watanabe (D) [46]: sx—saproxenes; es—eurysaprobes, sp—saprophiles. Saprobity groups according to Sládeček [42] (SAP): o—oligosaprob; o-b—oligo–beta–msosaprob; b-o—beta–oligosaprob; o-a—oligo-alpha–mesosaprob; b—beta–mesosaprob; b-a—beta–alpha–mesosaprob; a-o—alpha-oligosaprob; x-b, xeno-beta-mesosaprobiont; x-o, xeno-oligosaprobiont. Index S—Species-specific index saprobity. Nutrition type as nitrogen uptake metabolism (Van Dam et al., 1994) [64] (AUT-HET): ate—nitrogen–autotrophic taxa; ats—tolerating elevated concentrations of organically bound nitrogen; hne—facultatively nitrogen–heterotrophic taxa, needing periodically elevated concentrations of organically bound nitrogen. Trophic state indicators (Van Dam et al., 1994) [64] (TRO): om—oligo–mesotraphentic; m—mesotraphentic; me—meso–eutraphentic; e—eutraphentic; he—hypereutraphentic.
